# Thrombocytopenia and neutropenia in Epstein–Barr virus infectious mononucleosis: A retrospective cohort study

**DOI:** 10.17305/bb.2025.12998

**Published:** 2025-12-07

**Authors:** Patrick Naughton, James V Harte, Brigid Lucey, Frances Enright

**Affiliations:** 1 Department of Biological Sciences, Munster Technological University, Rossa Avenue, Bishopstown, Cork, Ireland; 2 Department of Haematology, Mercy University Hospital, Grenville Place, Cork, Ireland; 3 Department of Haematology, Cork University Hospital, Cork, Ireland; 4 Department of Paediatrics, Cork University Hospital, Cork, Ireland

**Keywords:** Atypical lymphocytes, Epstein–Barr virus, heterophile antibodies, infectious mononucleosis, Monospot test, neutropenia, thrombocytopenia

## Abstract

Thrombocytopenia and absolute neutropenia are recognized manifestations of Epstein–Barr virus infectious mononucleosis (EBV-IM). This study conducted a retrospective analysis of laboratory results from patients clinically suspected of having EBV-IM and tested over a two-year period (2018–2019) at a single testing center in Ireland, aiming to determine the prevalence of these hematological complications. A cohort of 51 confirmed acute EBV-IM cases was established, and the incidence of thrombocytopenia and absolute neutropenia within this group was assessed. These findings were then compared to the frequencies observed in non-acute EBV-IM patients, both with and without atypical lymphocytes. Among the 51 patients diagnosed with acute EBV-IM, 14% presented with thrombocytopenia and absolute neutropenia, including instances of severe cases. A comparable prevalence of these conditions was noted in non-acute EBV-IM patients with identifiable atypical lymphocytes; however, a significantly lower incidence was found in non-acute EBV-IM patients lacking atypical lymphocytes. These results suggest that thrombocytopenia and absolute neutropenia occur in patients with viral infections and are not exclusive to acute EBV-IM.

## Introduction

Infectious mononucleosis (IM) is primarily caused by human herpesvirus 4 (HHV-4) [1], more commonly known as Epstein–Barr virus (EBV) [2]. EBV is a lymphotropic, double-stranded, enveloped DNA oncogenic virus that belongs to the family *Orthoherpesviridae*, subfamily *Gammaherpesvirinae*, and genus *Lymphocryptovirus* [3, 4]; it is responsible for over 90% of all IM cases [5, 6].

The clinical presentations of primary EBV infection can range from asymptomatic cases to the classical triad of fever, pharyngitis, and cervical lymphadenopathy [7, 8]. Less common symptoms and complications include transient hepatitis, splenomegaly, malaise, nausea, palatal petechiae [9, 10], periorbital edema—the Hoagland sign [11–13]—, thrombocytopenia [14–16], and mild neutropenia [17, 18]. Primary infection with EBV typically occurs in infancy or early childhood and is generally subclinical; however, when contracted later in life, there is a higher likelihood of manifesting as EBV-associated IM (EBV-IM) [19–21].

EBV-IM is usually a self-limiting disease, with most symptoms resolving quickly, although fatigue may persist for several months following the onset of the condition [22]. When treatment is necessary, it primarily involves supportive care, including rest, hydration, and, in some cases, analgesics and antipyretics [5, 22].

Timely diagnosis of EBV-IM facilitates appropriate patient management and minimizes the need for additional exploratory tests and procedures in cases presenting with splenomegaly, lymphadenopathy, or suspected hematological conditions [3]. Identifying the viral source ensures that antibiotic treatment is reserved for bacterial infections, typically streptococcal complications, indicated by neutrophilia [23–25]. Unnecessary antibiotic treatment in EBV-IM cases can lead to an ampicillin-related rash in 70% to 100% of patients [24–27].

Diagnosing EBV-IM based solely on clinical symptoms is unreliable, as these symptoms can be similar to those of other bacterial and viral infections [3, 22], necessitating laboratory confirmation. Microscopic detection of a population of reactive (atypical) lymphocytes, namely cytotoxic T lymphocytes (CD8+ T cells) [22], often accompanied by relative or absolute lymphocytosis, suggests a viral etiology [7]. Historically, the presence of these atypical cells in clinically suggestive patients has directed investigations toward EBV-IM [28, 29]. Additionally, transient hepatitis, indicated by elevated liver enzymes, is non-specific but occurs in 80% to 90% of patients with EBV-IM, raising clinical suspicion [30–32].

Simple, inexpensive tests for detecting non-specific heterophile antibodies (HAs) remain popular for diagnosing EBV-IM due to their rapid, and easily demonstrable nature; however, they exhibit relatively high specificity but low sensitivity, particularly in children under five years of age [33]. HA tests are limited to typical HA-positive cases [34], and equivocal results should be confirmed with specific EBV serology [35]. In active cases of EBV-IM, expected serological results include the presence of acute IgM immunoglobulin, with or without subsequent IgG antibodies to the early EBV viral capsid antigen (VCA), and the absence of later-stage Epstein–Barr nuclear antigen (EBNA) IgG antibodies [36].

Hematological complications are reportedly common in patients with EBV-IM. Infection-related thrombocytopenia is generally caused by bone marrow suppression but can also be immune-mediated [37]. Thrombocytopenia is common during primary EBV infection, particularly in adult patients, where it is typically mild and transient [14]. More severe EBV-associated thrombocytopenia is a rare outcome and may necessitate specific management [16, 38]. Mild transient neutropenia has also been observed during the initial weeks of acute EBV infection [17, 18]; in less common cases, profound neutropenia and agranulocytosis may occur up to six weeks after the onset of initial symptoms [39].

Data on cytopenia in Irish patients with EBV-IM is scarce in the literature. Following a review of laboratory results from patients suspected of having and tested for EBV-IM over a two-year period at our testing center in Ireland, a retrospective case-control study was conducted to determine the frequency of thrombocytopenia and neutropenia in patients with EBV-IM. The findings were compared to two control groups: non-EBV-IM patients with atypical lymphocytes and non-EBV-IM patients without atypical lymphocytes.

## Materials and methods

### Design and setting

A retrospective analysis was conducted on the laboratory results of patients tested for EBV-IM at a single testing center in Ireland (MUH) over a two-year period from January 1, 2018, to December 31, 2019. The study design adhered to the Strengthening the Reporting of Observational Studies in Epidemiology (STROBE) checklist [40]. Most patients presented to the MUH Emergency Department, while a smaller number were referred for testing by their general practitioners. All patients were clinically unwell upon presentation and were suspected of having EBV-IM or another similar condition. Laboratory data were compiled from the Laboratory Information Management System at MUH.

**Figure 1.**
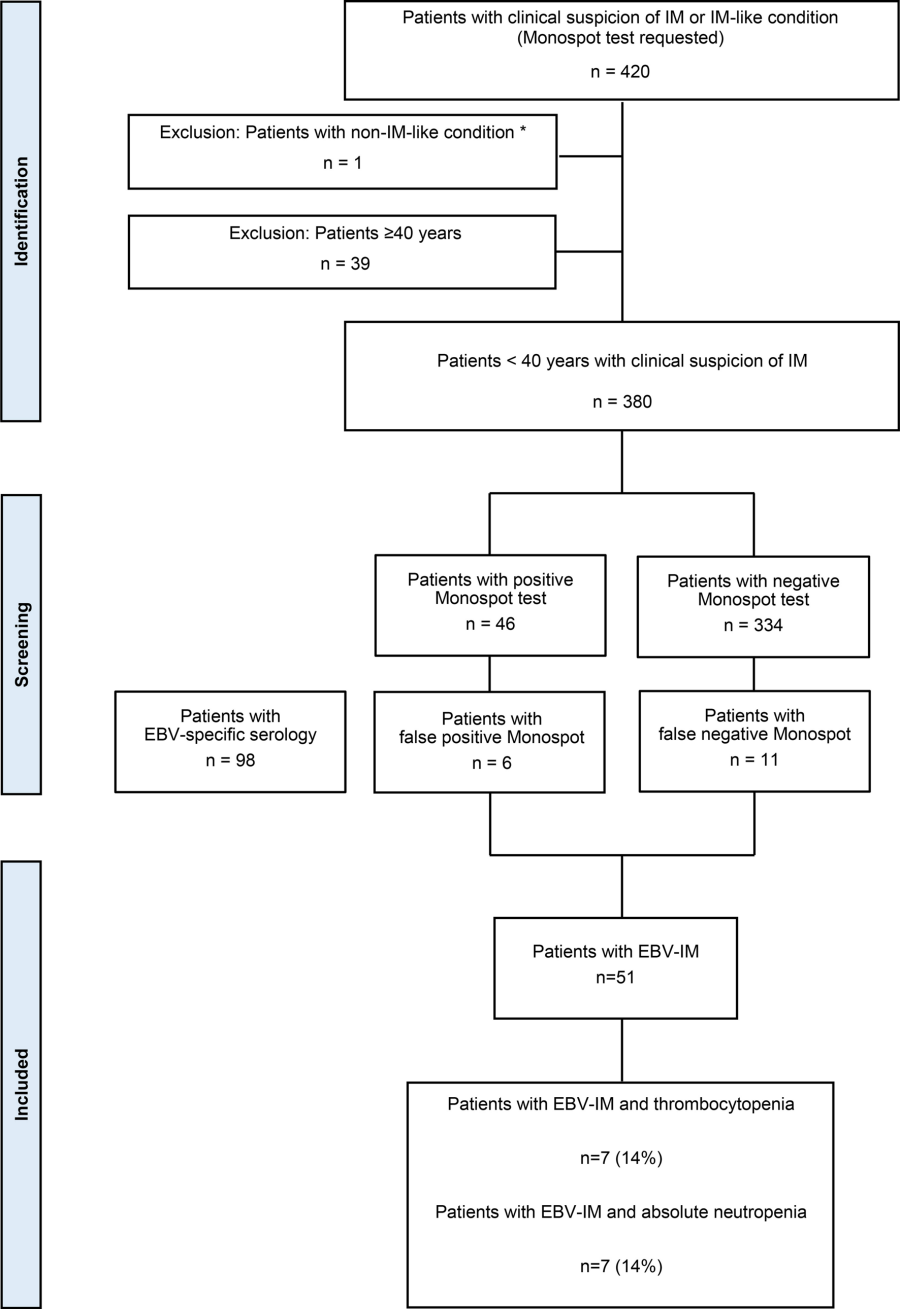
**Flowchart illustrating the frequency of thrombocytopenia and absolute neutropenia in patients diagnosed with EBV infectious mononucleosis (EBV-IM), following a retrospective analysis of patients investigated for infectious mononucleosis or other conditions resembling it at Mercy University Hospital, Ireland (MUH).** False positive and false negative Monospot tests were identified through the review of EBV serology. Abbreviations: EBV-IM: EBV infectious mononucleosis. *Patient diagnosed with hemophagocytic lymphohistiocytosis.

A total of 420 patients were identified with a suspicion of EBV-IM or a related condition during the two-year period, as indicated by a requested Monospot test. Given the rarity of the disease in older adults (≥40 years) [28, 34], only patients under 40 years of age were included in this study (*n* ═ 381). One additional patient was excluded due to a diagnosis of hemophagocytic lymphohistiocytosis (*n* ═ 1). The final dataset comprised 380 patients, categorized into three groups: a case group of confirmed EBV-IM patients (*n* ═ 51), a control group of non-acute EBV-IM patients with demonstrable atypical lymphocytes (*n* ═ 90), and a second control group of non-acute EBV-IM patients without atypical lymphocytes (*n* ═ 239). The frequency of thrombocytopenia and absolute neutropenia was analyzed and compared across all three groups (see Figure 1).

### Diagnosis of EBV-IM

Patients exhibiting clinical features indicative of IM were deemed positive for EBV-IM if they tested positive for HAs and demonstrated atypical lymphocytes [34]. Elevated absolute lymphocyte counts (ALCs) and raised liver function tests (LFTs) further supported the diagnosis (see Table 1). LFTs were considered elevated if any liver transaminases—namely aspartate aminotransferase (AST), alanine aminotransferase (ALT), or gamma-glutamyl transferase (GGT)—exceeded their normal upper reference range thresholds. Additionally, as EBV serology remains the gold standard for EBV-IM detection, patients with serological test results consistent with acute EBV-IM were classified as true EBV-IM cases, regardless of the presence of HAs. Specific EBV serology was conducted on a subset of patients (*n* ═ 108) at Cork University Hospital (CUH), Cork, and the Irish National Virus Reference Laboratory (NVRL), Dublin. Since only patients under 40 years of age were considered for this study, serological test results were available for 98 individuals in the retrospective cohort, with the remaining 10 tests performed on patients aged 40 years and older.

**Table 1:** Clinical laboratory results for patients tested for EBV-IM

	**Acute EBV-IM patients**	**Non-EBV-IM patients with atypical lymphocytes**	**Non-EBV-IM patients without atypical lymphocytes**
Total patients	51	90	239
Gender ratio	Male: 49% (25)	Male: 54% (49)	Male: 41% (99)
Median age [IQR]	17 [14, 19]	5 [1, 17]	17 [8, 24]
EBV serology performed	*n* ═ 24	*n* ═ 19	*n* ═ 55
EBV VCA IgM positive	100% (24/24)	21% (4/19)	9% (5/55)
EBV VCA IgG positive	63% (15/24)	42% (8/19)	58% (32/55)
EBNA positive	0% (0/24)	21% (4/19)	16% (9/55)
Monospot positive	78% (40/51)	1% (1/90)	2% (5/239)
Raised LFTs	82% (42/51)	53% (48/90)	20% (47/239)
Atypical lymphocytes (%)	98% (50/51)	100% (90/90)	0% (0/239)
Raised ALC	68% (35/51)	15% (14/90)	0.8% (2/239)
Thrombocytopenia	14% (7/51)	13% (12/90)	3% (8/239)
Absolute neutropenia	14% (7/51)	17% (15/90)	2% (5/239)

Results are presented as percentages (and corresponding values) or as medians along with IQR. LFTs were considered elevated if: ALT > 55 U/L, AST > 34 U/L, and GGT > 36 U/L (female) or GGT > 64 U/L (male). Normal reference ranges for ALC are age-dependent: 1 month to 1 year, 3--9 × 10^9^/L; 1–5 years, 4–10 × 10^9^/L; 5–10 years, 1.5–7.5 × 10^9^/L; 10–16 years, 1.6–6.5 × 10^9^/L; and >16 years, 0.9–3.2 × 10^9^/L. Results exceeding the upper threshold values were classified as elevated. Abbreviations: ALC: Absolute lymphocyte count; EBNA: Epstein–Barr nuclear antigen; EBV: Epstein–Barr virus; EBV VCA: EBV viral capsid antigen; IQR: Interquartile range; LFTs: Liver function tests.

### Laboratory testing

The qualitative detection of HAs in patients was performed using the Clearview^®^ IM II Monospot test (Abbott Rapid Dx International Ltd., Parkmore East Business Park, Ballybrit, Galway, Ireland), following the manufacturer’s instructions. Peripheral blood analyses were conducted using a Siemens Advia 2120i full blood count and differential analyzer (Siemens Ltd., 8-11 Slaney Road, Dublin 11, Ireland). Peripheral blood samples flagged for abnormal results (e.g., blast or atypical lymphocyte flags, absolute lymphocytosis, absolute thrombocytopenia [<100 × 10^9^/L], and/or a large unstained cell population >0.45 × 10^9^/L) underwent further morphological investigation through blood smear preparation, staining, and examination by medical scientists at MUH. LFTs were conducted in the Biochemistry Department of MUH, utilizing the Abbott Alinity analyzer (Abbott Ireland, Diagnostics Division, Lisnamuck, Longford, Ireland). Elevations in any liver enzyme levels beyond the normal upper reference range thresholds were considered biomarkers of liver damage and/or hepatitis: ALT > 55 U/L, AST > 34 U/L, and GGT > 36 U/L (female) or GGT > 64 U/L (male).

Patients with platelet counts <150 × 10^9^/L were classified as thrombocytopenic [37]. Mild thrombocytopenia was defined as platelet counts between 100 and 150 × 10^9^/L, moderate thrombocytopenia as counts between 50 and 100 × 10^9^/L, and severe thrombocytopenia as counts below 50 × 10^9^/L. Low platelet counts were verified through blood smear examination to exclude pseudothrombocytopenia [14, 37].

Patients with an absolute neutrophil count below the age-dependent thresholds were classified as neutropenic: <1.5 × 10^9^/L for ages 1 month to 5 years; <2.0 × 10^9^/L for ages 5–10 years; <2.5 × 10^9^/L for ages 10–16 years; and <1.4 × 10^9^/L for individuals aged 16 years or older. These age-dependent thresholds represent the lower limits of the respective reference ranges, which were validated internally (data unpublished).

### Ethical statement

Full ethical approval for this study was granted by the Cork Research Ethics Committee, Ireland (ECM 4(r) 08/12/18 and ECM 4(s) 07/05/19). Neither patients nor the public were involved in the design, conduct, reporting, or dissemination plans of this research.

### Statistical analysis

Data were compiled using Microsoft Excel 2007 software. No *a priori* power calculation was performed prior to the study’s initiation. Results are presented as percentages (with corresponding values) or as medians (and interquartile ranges). Percentages were rounded to the nearest whole number. Statistical analyses were conducted using GraphPad Prism (Version 10.4.1). Confidence intervals (CIs) for categorical proportions were calculated using the modified Wald method [41]; statistically significant differences for categorical proportions were assessed using Fisher’s exact test [42]. A two-sided *P* value threshold of less than 0.05 was considered indicative of statistical significance.

## Results

During the retrospective study period, 46 patients tested positive for HAs via the Monospot test: 30 (65%) presented with atypical lymphocytes and lymphocytosis; 10 (22%) exhibited atypical lymphocytes without lymphocytosis; and 6 (13%) had neither atypical lymphocytes nor lymphocytosis.

Additional EBV serology was available for 17 patients with positive Monospot test results and 91 patients with negative results for HAs. Based on serological testing, 6 of the positive Monospot results were classified as false positives: 2 patients had evidence of post-acute infection, indicated by positivity for EBNA IgG antibodies; one patient exhibited 1% atypical lymphocytes upon blood film analysis alongside bacteria-associated neutrophilia; other false positive patients lacked atypical lymphocytes, had normal ALCs, and LFTs were within normal ranges. Furthermore, 11 negative Monospot results were deemed false negatives, as all 11 patients demonstrated specific EBV serology consistent with active infection, indicated by positivity for EBV VCA IgM in the absence of EBNA IgG, and displayed atypical lymphocytes with elevated LFTs.

In total, 51 patients with confirmed EBV-IM were identified: 27 cases were confirmed through positive Monospot (HA) test results in the presence of demonstrable atypical lymphocytes, while an additional 24 cases were validated by specific EBV serology for EBV VCA IgM (Figure 1 and Table 1).

The remaining patients (*n* < 40 years) were categorized as either non-EBV-IM with atypical lymphocytes (*n* ═ 90) or non-EBV-IM without atypical lymphocytes (*n* ═ 239). No further clinical information was available for these patients through the Laboratory Information Management System at the MUH.

Thrombocytopenia was observed in 14% (*n* ═ 7; 95% CI: 7%--26%) of patients with acute EBV-IM (Table 1). Most of these patients exhibited mild thrombocytopenia, with platelet counts ranging from 103–135 × 10^9^/L; however, one patient, a 15-year-old male, exhibited severe thrombocytopenia with a platelet count of 12 × 10^9^/L. Age stratification revealed no statistically significant difference in the frequency of thrombocytopenia between patients aged < 18 years (*n* ═ 29) and those aged ≥ 18 years (*n* ═ 22) with acute EBV-IM, despite a trend toward increased prevalence in adult patients (Table 2): thrombocytopenia was present in 10% (*n* ═ 3; 95% CI: 3%–30%) of patients aged < 18 years and in 18% (*n* ═ 4; 95% CI: 7%–40%) of patients aged ≥ 18 years (thrombocytopenia *P* value: 0.447).

**Table 2:** Age-stratified frequency of thrombocytopenia and neutropenia in patients tested for EBV-IM

	**Acute EBV-IM patients < 18 years**	**Acute EBV-IM patients ≥ 18 years**
Total patients	*N* ═ 29	*N* ═ 22
Gender ratio	Male: 52% (*n* ═ 15)	Male: 46% (*n* ═ 10)
Median age [IQR]	14 [4, 16]	19 [19, 21]
Thrombocytopenia	10% (3/29)	18% (4/22)
Absolute neutropenia	20% (6/29)	5% (1/22)

The results of the age-stratified analysis are similarly presented as percentages (and corresponding values) or as medians along with IQR. Abbreviation: IQR: Interquartile range.

Additionally, moderate neutropenia was observed in 14% (*n* ═ 7; 95% CI: 7%–26%) of patients with acute EBV-IM (Table 1). Age stratification indicated no statistically significant difference in the frequency of neutropenia between patients aged < 18 years (*n* ═ 29) and those aged ≥ 18 years (*n* ═ 22) with acute EBV-IM, despite a trend toward increased prevalence in pediatric patients (Table 2): neutropenia was present in 20% (*n* ═ 6; 95% CI: 10%–39%) of patients aged < 18 years and in 5% (*n* ═ 1; 95% CI: 0%–24%) of patients aged ≥ 18 years (neutropenia *P* value: 0.124).

It is important to note that patients presenting with thrombocytopenia did not exhibit concomitant neutropenia, and those with neutropenia did not show concomitant thrombocytopenia. These findings indicate that quantitative changes in platelets and neutrophils may occur as mutually exclusive events.

No statistically significant differences were observed in the frequency of thrombocytopenia or neutropenia among patients with acute EBV-IM compared to those without EBV-IM but with atypical lymphocytes (thrombocytopenia *P* value: >0.999; neutropenia *P* value: 0.810). However, when compared to patients without acute EBV-IM lacking atypical lymphocytes, the frequency of both thrombocytopenia (*P* value: 0.007) and neutropenia (*P* value: 0.001) was significantly greater in patients with acute EBV-IM, as well as in patients without acute EBV-IM with atypical lymphocytes (thrombocytopenia *P* value: <0.001; neutropenia *P* value: <0.001). This suggests that patients presenting with atypical lymphocytes may be at a higher risk of developing thrombocytopenia and neutropenia than those without atypical lymphocytes.

In the acute EBV-IM cohort, 47% (24/51) underwent EBV serology testing. Consistent with the cohort identified through the detection of HAs, thrombocytopenia was noted in 17% (*n* ═ 4; 95% CI: 7%–33%) and neutropenia in another 17% (*n* ═ 4; 95% CI: 7%–33%) of serologically tested patients (Table 3). Furthermore, no statistical differences in cytopenia frequency were found between patients with atypical lymphocytes, regardless of the presence of acute EBV-IM (thrombocytopenia *P* value: 1.000; neutropenia *P* value: 0.4674). However, the frequency of cytopenia was generally higher in both the patients with atypical lymphocytes and acute EBV-IM (thrombocytopenia *P* value: 0.066; neutropenia *P* value: 0.028) and in those with atypical lymphocytes without acute EBV-IM (thrombocytopenia *P* value: 0.1033; neutropenia *P* value: 0.004), with statistically significant differences observed particularly for neutropenia (Table 3).

**Table 3:** Clinical laboratory results for patients tested for EBV-IM, restricted to those with serological testing

	**Acute EBV-IM patients**	**Non-EBV-IM patients with atypical lymphocytes**	**Non-EBV-IM patients without atypical lymphocytes**
Total patients	*N* ═ 24	*N* ═ 19	*N* ═ 55
Gender ratio	Male: 46% (*n* ═ 11)	Male: 37% (*n* ═ 7)	Male: 31% (*n* ═ 17)
Median age [IQR]	15 [4, 19]	1 [1, 13]	16 [8, 26]
Monospot positive	54% (13/24)	5% (1/19)	5% (3/55)
Raised LFTs	92% (22/24)	53% (10/19)	22% (12/55)
Atypical lymphocytes (%)	96% (23/24)	100% (19/19)	0% (0/55)
Raised ALC	54% (13/24)	10% (2/19)	0% (0/55)
Thrombocytopenia	17% (4/24)	20% (3/19)	4% (2/55)
Absolute neutropenia	17% (4/24)	26% (5/19)	2% (1/55)

The results of the serology-restricted sensitivity analysis are again presented as percentages (and corresponding values) or as medians with IQR. LFTs were deemed elevated if: ALT > 55 U/L, AST > 34 U/L, and GGT > 36 U/L (female) or GGT > 64 U/L (male). Abbreviations: ALC: Absolute lymphocyte count; IQR: Interquartile range; LFTs: Liver function tests.

## Discussion

This retrospective study assessed the frequency of neutropenia and thrombocytopenia in a cohort of patients diagnosed with EBV-IM at an Irish center across the first four decades of life. Most primary EBV infections occur during the first two decades; however, a smaller proportion of individuals contract primary EBV infection later in life, especially in developed countries, where it is more likely to result in symptomatic EBV-IM [22]. The diagnosis of EBV-IM was based on a combination of positive HA test results (Monospot test) and demonstrable atypical lymphocytes (*n* ═ 27), alongside positive EBV serology for EBV-IM (*n* ═ 24).

Among the 51 acute EBV-IM cases, a modest frequency of thrombocytopenia (14%; 95% CI: 7%–26%) and neutropenia (14%; 95% CI: 7%–26%) was recorded. The observed prevalence of thrombocytopenia in our retrospective cohort was lower than the widely cited frequency of 25%–50% reported by Carter [43], while the prevalence of neutropenia was slightly higher than that reported in previous retrospective cohort studies [44, 45]. Notably, earlier studies have indicated a potential age-dependent variability in the prevalence of thrombocytopenia in acute EBV-IM: in patients under 18 years, Gao et al. [45] reported thrombocytopenia in 5% and neutropenia in 4%, while González Saldaña et al. [46] noted thrombocytopenia in 7%, and Son and Shin [44] reported thrombocytopenia in 10% and absolute neutropenia in 8.6%. More recently, Páez-Guillán et al. [14] found thrombocytopenia in up to 30% of patients aged 15 years and older. Although differences in the frequency of thrombocytopenia between pediatric and adult patients at our center did not reach statistical significance, trends suggest a potentially greater risk in adult patients (18%; 95% CI: 7%–40%) compared to pediatric patients under 18 years (10%; 95% CI: 3%–30%). In contrast, while differences in the frequency of neutropenia between pediatric and adult patients also did not achieve statistical significance, trends indicate a potentially higher risk of neutropenia in pediatric patients (20%; 95% CI: 10%–39%) compared to adult patients (5%; 95% CI: 0%–24%). The limitations of the small sample size hinder definitive conclusions; however, further studies focusing on age-stratified cohorts may be warranted based on existing literature [45, 46].

Interestingly, we observed that non-acute EBV-IM patients exhibiting atypical lymphocytes, potentially indicative of resolving viral infection or other IM-like conditions, displayed frequencies of thrombocytopenia and neutropenia comparable to those in the true acute EBV-IM cohort. In contrast, non-acute EBV-IM patients without detectable atypical lymphocytes demonstrated significantly lower frequencies of both thrombocytopenia and neutropenia. Collectively, these findings suggest that the prevalence of thrombocytopenia and neutropenia in patients with EBV-IM and similar conditions may be analogous, indicating that these hematological changes are not exclusive to EBV infections. Specifically, the incidence of thrombocytopenia and neutropenia is markedly higher in clinically unwell patients exhibiting atypical lymphocytes compared to those who do not.

The findings of this retrospective study are constrained by the limited sample size, derived from a single testing center. No *a priori* power calculation was performed, and we recognize that this limitation introduces a certain degree of imprecision. Furthermore, the retrospective nature of the study meant that not all patients underwent the same breadth and type of testing, as laboratory test requests were largely at the discretion of the clinical team. Specific requests for EBV serology were not standardized and were conducted only on a subset of the total patient population. Consequently, we cannot exclude the possibility that some patients, regardless of the presence of atypical lymphocytes, may have been entirely negative for EBV-IM despite negative Monospot test results. Lastly, since data were collected from the Laboratory Information Management System, clinical data was unfortunately unavailable throughout the study, and our ethical approval did not encompass access to patients’ clinical files.

## Conclusion

This study indicates that patients with EBV-IM in Ireland exhibit thrombocytopenia and neutropenia, albeit at a relatively low frequency. Clinicians and medical scientists should consider EBV as a potential cause of cytopenia in patients with atypical lymphocytes. Notably, our findings also reveal that patients with viral or IM-like conditions—characterized by the presence of atypical lymphocytes—exhibit a higher frequency of thrombocytopenia and neutropenia compared to patients lacking atypical lymphocytes, with no significant difference observed between those diagnosed with acute EBV-IM and those with other non-specific EBV-IM-like conditions. These observations underscore the need for further investigation in larger clinical cohorts.

## Data Availability

The data that support the findings of this study are available on request from the corresponding author, Patrick Naughton.
